# Helical TomoTherapy Total Lymphoid Irradiation and Hematopoietic Cell Transplantation for Kidney Transplant Tolerance in Rhesus Macaques

**DOI:** 10.3389/ti.2023.11279

**Published:** 2023-06-22

**Authors:** Dixon B. Kaufman, Lisa J. Forrest, John Fechner, Jennifer Post, Jennifer Coonen, Lynn D. Haynes, W. John Haynes, Neil Christensen, Weixiong Zhong, Christopher J. Little, Anthony D’Alessandro, Luis Fernandez, Kevin Brunner, Kent Jensen, William J. Burlingham, Peiman Hematti, Samuel Strober

**Affiliations:** ^1^ Department of Surgery, University of Wisconsin, Madison, WI, United States; ^2^ School of Veternary Medicine, University of Wisconsin, Madison, WI, United States; ^3^ Wisconsin National Primate Research Center, University of Wisconsin, Madison, WI, United States; ^4^ Department of Pathology, University of Wisconsin, Madison, WI, United States; ^5^ Department of Medicine, Stanford University, Palo Alto, CA, United States; ^6^ Department of Medicine, University of Wisconsin, Madison, WI, United States

**Keywords:** kidney transplantation, tolerance induction, chimerism, hematopoietic cells, TomoTherapy

## Abstract

Development of a post-transplant kidney transplant tolerance induction protocol involving a novel total lymphoid irradiation (TLI) conditioning method in a rhesus macaque model is described. We examined the feasibility of acheiving tolerance to MHC 1-haplotype matched kidney transplants by establishing a mixed chimeric state with infusion of donor hematopoietic cells (HC) using TomoTherapy TLI. The chimeric state was hypothesized to permit the elimination of all immunosuppressive (IS) medications while preserving allograft function long-term without development of graft-versus-host-disease (GVHD) or rejection. An experimental group of 11 renal transplant recipients received the tolerance induction protocol and outcomes were compared to a control group (*n* = 7) that received the same conditioning but without donor HC infusion. Development of mixed chimerism and operational tolerance was accomplished in two recipients in the experimental group. Both recipients were withdrawn from all IS and continued to maintain normal renal allograft function for 4 years without rejection or GVHD. None of the animals in the control group achieved tolerance when IS was eliminated. This novel experimental model demonstrated the feasibility for inducing of long-term operational tolerance when mixed chimerism is achieved using a TLI post-transplant conditioning protocol in 1-haplotype matched non-human primate recipients of combined kidney and HC transplantation.

## Graphical Abstract

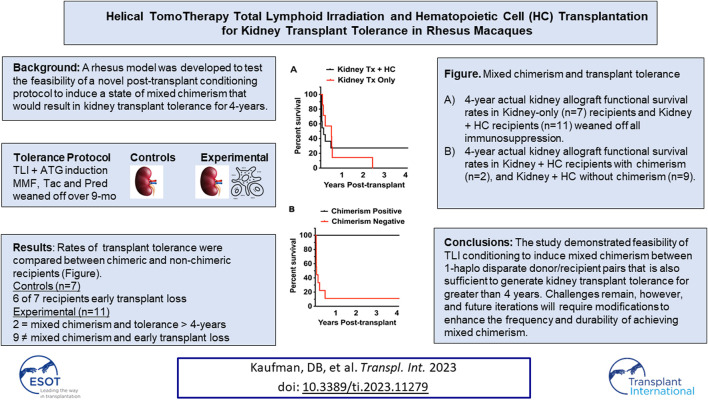

## Introduction

Complete elimination of immunosuppressive (IS) medications in solid organ transplant recipients results in allograft rejection and graft loss unless host immune tolerance to the donor organ is induced. A promising development in allograft tolerance induction is the creation of a chimeric immune state within the transplant recipient comprised of both host and donor immune cellular elements [1–15]. This can be accomplished by application of a conditioning protocol to the recipient followed by hematopoietic cell (HC) transplantation from the organ donor into recipient. Establishing a chimeric state allows IS elimination without organ allograft loss from rejection [1–16].

Of particular relevance to this report is the success in human studies applying a post-transplant, non-myeloablative, total lymphoid irradiation (TLI) and anti-thymocyte globulin (ATG) conditioning protocol to achieve engraftment of donor HCs that produce a stable mixed chimeric state without graft versus host disease (GVHD) [2–8, 17–19]. The development of a chimeric state permitted elimination of all IS without inducing cellular- or antibody-mediated immune injury among HLA-identical living related pairs [5–7].

The non-human primate (NHP) model described in this study replicated the human protocol. The purpose of this translational study was to inform future clinical development of the TLI tolerance induction protocol as applied to donor/recipient pairs with greater MHC disparity than the current clinical studies between HLA-identical transplants. Human studies involving TLI-induced tolerance had not previously been conducted in recipients of 1-haplo matched living donor kidneys.

This pre-clinical primate study examined the feasibility of a novel post-transplant, non-myeloablative conditioning protocol comprised of helical tomotherapy-based TLI (TomoTherapy TLI) and ATG in conjunction with donor peripheral blood mobilized HC infusion to induce a state of mixed chimerism in a rhesus macaque 1-haplotype MHC matched living related kidney transplant model [20]. We hypothesized that the chimeric state would result in operational tolerance for 4-years and without GVHD. We evaluated the extent to which this tolerance induction protocol induced recipient immunomodulation, the frequency and durability of achieving mixed chimerism, rates of GVHD and rejection, generation of Class I and II donor-specific antibody (DSA), CMV reactivation, and the rate of long-term (4-year) kidney transplant operational tolerance.

## Materials and Methods

### Animals and Determination of Donor-Recipient Pairs

Male and female rhesus macaques were obtained from the NIAID colony maintained by Alpha Genesis Inc. (Yemassee, SC) (Table 1). All animals were treated in accordance with the 8th edition of the Guide for the Care and Use of Laboratory Animals published by National Research Council and the procedures and protocol were approved by the University of Wisconsin-Madison institutional animal care and use committee.

**TABLE 1:** Hematopoeitic cell therapy and transplant outcome by experimental group.

ID	Weight (kg)	Sex	Relationship	MHC type	TNC (x 106 kg)	CD3^+^ cells (x 106 kg)	CD34^+^ cells (x 106 kg)	Kidney survival (days)	Outcome
Kidney Transplant Only									
C1	5.7	F	Mother/Child	A016, A003, B024a, B017a, DR35, DR04a	-	-	-	45	AMR
C1-D	11.4	F		A016, A001, B024a, B043b, DR35, DR03f					
C2	11.0	M	Mother/Child	A004, A008, B012b, B047a, DR04a, DR09a	-	-	-	188	Rejection/volvulus
C2-D	5.3	F		A004, A019, B012b, B048, DR04a, DR03f					
C3	6.4	F	Mother/Child	A004, A023, B017a, B012b, DR11c, DR15ab	-	-	-	1089	CR/AMR
C3-D	6.6	F		A004, A023, B017a, B055, DR11c, DR04a					
C4	4.9	F	Siblings	A001, A008, B024a, B012a, DR15a/b, DR03f	-	-	-	199	PTLD
C4-D	5.5	M		A001, A008, B024a, B069b, DR15a/b, DR04a					
C5	6.1	M	Siblings	A008, A004, B-unk, B001a, DR05a, DR04a	-	-	-	29	Failure to Thrive, AKI
C5-D	5.1	F		A008, A006, B-unk, B043a, DR05a, DR03f					
C6	5.7	M	Mother/Child	A004, A002a, B002, B012a, DR06, DR03f	-	-	-	79	AMR
C6-D	7.9	F		A004, A004, B002, B028, DR06, DR14a					
C7	5.2	M	Mother/Child	A018a, A002a, B002, B012a, DR27b, DR03f	-	-	-	185	Peritubular capillaritis
C7-D	9.6	F		A018a, A004, B002, B048, DR27b, DR15c					
Kidney + Hematopoietic Cell Transplant									
E1	5.7	M	Mother/Child	A025, A023, B017a, B012b, DR16, DR15c	440	57	2.7	>3416	Survival
E1-D	6.7	F		A025, A004, B017a, B048, DR16, DR15c					
E2	9.0	M	Mother/Child	A008, A224a, B055, B001a, DR06, DR03a	1250	163	15.4	176	AMR
E2-D	10.0	F		A008, A004, B055, B012b, DR06, DR03f					
E3	10.0	M	Mother/Child	A002a, A065, B017a, B003a, DR03f, DR04c	1111	479	4.1	19	Engraftment syndrome
E3-D	7.2	F		A002a, A006, B017a, B047a					
E4	5.4	F	Mother/Child	A001, A004, B012a, B012b, DR04a, DR05a	1464	173	6.6	18	Engraftment syndrome
E4-D	7.2	F		A001, A001, B012a, B043b, DR04a, DR03f					
E5	6.2	F	Siblings	A006, A004, B001a, B001a, DR03a, DR04a	400	176	1.5	1652	CR/AMR
E5-D	9.2	M		A006, A006, B001a, B043a, DR03a, DR03f					
E6	6.1	M	Mother/Child	A004, A004, B015a, B055, DR03a, DR03e	1900	617	4.5	79	Infection (Parvovirus)
E6-D	7.0	F		A004, A224a, B015a, B045a, DR03a, DR16					
E7	10.9	M	Siblings	A016, A002a, B001a, B015a, DR13a, DR15a	320	157	4.1	24	Infection (CMV)
E7-D	5.5	M		A016, A001, B001a, B001a, DR13a, DR01c					
E8	5.2	F	Siblings	A002a, A002a, B012a, B012a, DR03f, DR03f	680	97	0.7	16	Engraftment syndrome
E8-D	9.3	M		A002a, A002a, B012a, B001a, DR03f, DR02					
E9	10.6	M	Mother/Child	A026, A004, B001a, B001a, DR16, DR04a	106	12.5	1	50	AMR
E9-D	9.8	F		A026, A004, B001a, B012b, DR16, DR04a					
E10	6.1	F	Mother/Child	A004, A028, B012a, B012a, DR03f, DR04a	1144	356	4	18	Engraftment syndrome
E10-D	8.9	F		A004, A028, B012a, B048, DR03f, DR01a					
E11	5.7	M	Siblings	A001, A004, B001a, B002, DR01c, DR06	1373	183	8.4	1814	CR/AMR
E11-D	8.9	M		A001, A016, B001a, B001a, DR01c, DR13a					

Animals were 4.9–10.0 kg and 3.3–12.1-years-old at the time of the procedures. Both males and females were used as donors and recipients. MHC Class I and Class II typing of recipient and donor animals were performed by the WNPRC Genetics Services Unit as previously described [21, 22]. These MHC haplotyping results, along with pedigree analysis, were used to determine appropriate 1-haplotype matched donor/recipient pairs for each transplant**.** The kidney allografts came from blood group compatible, MHC 1-haplotype matched donors (either full siblings or maternal donors). Anti-donor T and B cell flow cytometric crossmatches were negative in all recipients pre-transplant.

### Investigative Protocol Design

**FIGURE 1:**
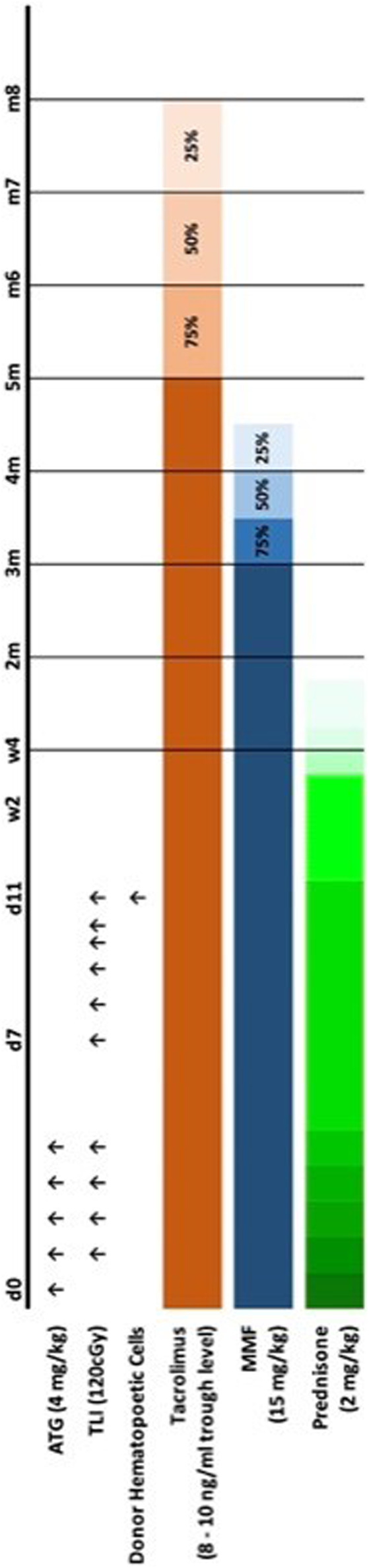
TomoTherapy TLI/ATG tolerance induction protocol and maintenance immunosuppression weaning.

Rates of kidney transplant tolerance were compared between the controls (*n* = 7, no HC infusion) and the experimental (*n* = 11, HC infusion) groups. All recipients received the same post-transplant conditioning protocol that included TLI and ATG. All animals had the IS eliminated according to the same tapering schedule (Figure 1). Seven animals in the control group received kidney transplants alone, and 11 animals in the experimental group received a combined kidney transplant plus the mobilized peripheral HC product. We monitored for donor immune cell engraftment in the experimental (kidney + HC) group by determining the frequency and durability of the chimeric state within multiple immunologic lineages.

### Conditioning and Immunosuppression Protocol

A novel method of TLI delivery using helical TomoTherapy (TomoTherapy TLI) was implemented as previously reported [20]. Briefly, TomoTherapy TLI was administered to the recipients on post-operative day 1 following kidney transplantation. The animals received 120cGy radiation divided over 10 doses similar to the dosing protocol in human trials (Figure 1). Donor peripheral blood mobilized HCs were collected as previously described for infusions occurring on post-transplant day 11 [23]. The native kidneys were removed immediately following renal allo-transplantation and submitted to pathology for documentation of their removal.

### Induction and Maintenance Immunosuppression

The immunosuppression protocol is illustrated in Figure 1. All rhesus transplant recipients received induction therapy consisting of five consecutive daily doses of 4 mg/kg anti-thymocyte globulin [ATG - either Thymoglobulin®, or the rhesus specific ATG generated by the NIH Nonhuman Primate Reagent Resource (supported by HHSN272200900037C and OD010976)] beginning at the time of kidney transplantation (day 0). Animals also received methylprednisolone (2 mg/kg), acetaminophen (5 mg/kg), and diphenhydramine (1 mg/kg) intravenously immediately prior to ATG infusion to minimize adverse reactions to the medication. Post-transplant maintenance IS consisted of corticosteroids, mycophenolate mofetil (MMF) administered orally at 15 mg/kg BID, and tacrolimus administered intramuscularly at 0.03 mg/kg BID with levels monitored 1–2 times per week to maintain a 12-hour trough level of 8–10 ng/mL.

### Immunosuppression Elimination Protocol

All recipients underwent IS withdrawal as planned (Figure 1). The corticosteroids were weaned and eliminated over post-transplant week 1. Beginning on day 90 post-transplant MMF was tapered 25% every 2 weeks. Subsequently, 2 weeks after completing the MMF withdrawal (month 4.5), the tacrolimus taper began by reducing the dosage approximately 25% each month to achieve decreasing trough levels of 6–8 ng/mL (month 5), 4–6 ng/mL (month 6), and 2–4 ng/mL (month 7) before being eliminated by month 8. Thereafter no IS therapy was administered to the recipient, nor was acute rejection treated with adjuvant IS at any point during the protocol.

### Helical TomoTherapy for Total Lymphoid Irradiation

Details of the Helical TomoTherapy TLI protocol has been previously reported [20]. TLI was planned and delivered by imaged-guided, intensity modulated helical TomoTherapy (TomoTherapy Hi-Art II, Accuray Inc, Sunnyvale CA). The total lymphoid target included the inguinal, iliac, sublumbar, para-aortic, axillary and mandibular lymph nodes, as well as the spleen and anterior mediastinal/thymic tissues.

### Donor Peripheral Blood Mobilized CD34^+^ Hematopoietic Cell and CD3^+^ T-cell Collection

Details of the apheresis procedure has been previously reported [23]. Briefly, donor animals received G-CSF (50 mcg/kg/d) for four consecutive days prior to, and on the day of, apheresis. In addition, one dose of plerixafor (Mozobil) (1 mg/kg) ∼3 h prior to apheresis of peripheral blood hematopoietic cells was administered. Flow cytometric analyses were performed on the apheresis product before freezing and prior to infusion for determination of frequency and total numbers of donor peripheral blood CD34^+^ and CD3^+^ cells.

### Chimerism Assessment

The chimeric state was assessed in the recipients by measuring the proportion of donor cells and the immunologic subset of each subtype. Chimerism was measured in the peripheral blood and bone marrow compartments of the recipient using a PCR-based assay. DNA was purified from peripheral whole blood cells (Qiagen Blood kit; with minor modifications) to be used as template for PCR with fluorescent-labeled primers specific to microsatellite loci (Dr. Cecilia Penedo, Veterinary Genetics Laboratory, UC Davis, CA) [24, 25]. To assess chimerism in specific cellular compartments, lymphocytes were fractionated from granulocytes using Lymphocyte Separation Media (Mediatech, Manassas, VA). DNA was then purified from the granulocyte fraction and MACS (Miltenyi Biotec, Auburn, CA) separated subsets of CD3^+^ T cells, CD20^+^ B cells, and non-CD3/CD20 cells (commercial human separation kits with known NHP cross-reactivity were utilized). The limit of detection was approximately 2%–4% by STR analysis.

### Donor Specific Antibody Monitoring

A standard flow cytometry-based assay was utilized to measure pre- and post-transplant donor-specific antibodies (DSA) in recipients. Plasma isolated from peripheral blood before and serially after transplantation was diluted 1:25 and incubated separately with donor (experimental) and recipient (control) PBMC. Cells were washed and stained for T cells (CD3) and B cells (CD20), as well as with the anti-rhesus IgG1/IgG3 antibody, 1B3-FITC (NHP-Reagent Resource, Boston, MA). After incubation and washing, cells were fixed and flow was acquired using an Accuri C6 flow cytometer (BD biosciences, San Jose, CA). Data was analyzed using FlowJo software (FlowJo LLC, Ashland, OR). Post-transplant FITC MFI shift on donor T (expressing only MHC class I) or B cells (expressing both MHC class I and II) of more than 2-fold compared to pre-transplant plasma or self-cell controls was considered positive for antibody.

### Rhesus Cytomegalovirus (rhCMV) Monitoring

Plasma samples were analyzed for evidence of rhCMV reactivation by detection of rhCMV DNA. The plasma samples were purified on a QIASymphony DNA Extraction System according to manufacturer protocols (QIAGEN) [26, 27]. DNA from a volume of 350 μL of plasma were extracted and eluted in an equivalent volume so that there was no dilution or concentration of any viral DNA. RhCMV genome copy numbers were quantified by qPCR assay methods based on a gB primer/probe set with a broad linear range of sensitivity [27]. Samples (5 μL of purified DNA) were analyzed in triplicate with a QuantStudio Flex 6 sequence detection system (Applied Biosystems). Each qPCR plate contained a 10-fold serial dilution of a CsCl-purified plasmid standard (10^6^–10^0^ plasmid molecules per 5 mL) containing the amplicon in order to provide a standard curve for each plate. Plate results were considered valid only if efficiencies were 90%–110%. qPCR results were normalized to RhCMV genomes/mL of plasma.

### Immune Suppressive Function of Recipient Myeloid-Derived Suppressor Cells

Ficoll gradient enriched PBMCs were labelled with CFSE according to manufacturer protocols (Invitrogen/Thermo Fisher Scientific). T cells were enriched from CFSE labeled cells using a negative selection kit that eliminates all non-T cells (Stem Cell Technologies) according to manufacturer’s protocol. Recipient Lin^−^CD11b^hi^DR^lo^ MDSCs, Lin^−^CD11b^hi^DR^hi^CD14^hi^ monocytes, and Lin^−^CD11b^lo^DR^hi^CD11c^+^CD14^−^ DCs were sorted on a FACSAria sorter (BD Biosciences) to >90% purity to be added to cultures to be assayed for suppressor activity (Supplementary Figure S1). Following enrichment and labeling with CFSE, T cells were resuspended to 1 × 10^6^ cells/mL in complete IMDM medium, and placed in 96 well U bottom tissue culture plates (1 × 10^5^/well) for 5 days at 37°C in 5% CO_2_/air either alone or in the presence of anti-CD2/CD3/CD28 NHP T cell activation microbeads (Miltenyi Biotec). These anti-CD2/CD3/CD28 T cell beads were added at a ratio of one bead per eight T cells. MDSCs, monocytes, or DCs (5 × 10^4^ cells/well, 1 suppressor cell per 2 T cells) were added to parallel wells containing T cells plus beads for co-culture as described above. Cultured cells were stained with fluorochrome labeled antibodies specific to CD3, CD4, and CD8 as well as T cell activation/differentiation markers. CFSE was measured to determine T cell proliferation (CFSE low indicating replicative cycles). Suppression of proliferation was calculated using the following formula: 1-[(%CFSEdim T cells from T+beads+suppressor cell co-cultures)/(%CFSEdim T cells from T+beads co-cultures)] x100.

## Results

### Outcomes and Association of Mixed Chimerism With Long-Term Graft Acceptance

**FIGURE 2:**
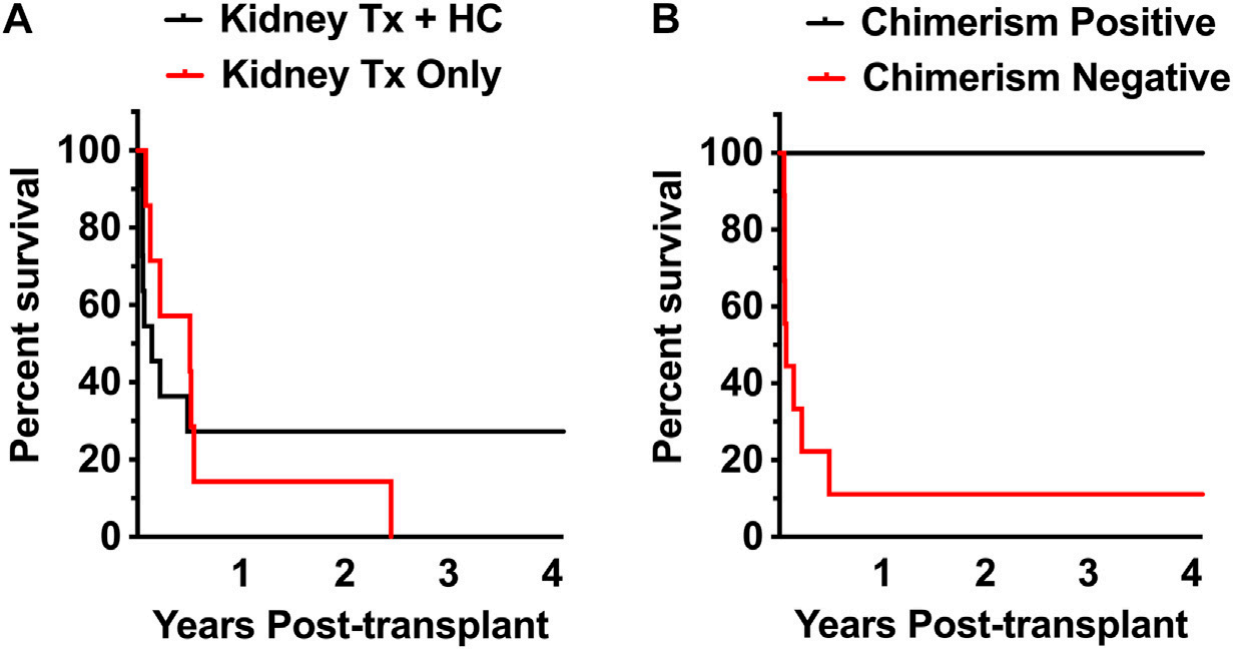
Peripheral donor cell chimerism is associated with kidney allograft tolerance. **(A)** 4-year actual kidney allograft functional survival rates in Kidney-only (*n* = 7) recipients and Kidney Tx + HC recipients (*n* = 11) weaned off all immunosuppression. **(B)** 4-year actual kidney allograft functional survival rates in Kidney Tx + HC recipients without chimerism (*n* = 9), and Kidney Tx + HC with chimerism recipients (*n* = 2).

In the control group of kidney transplant-only transplants, 6 of 7 recipients lost renal allograft function early during the period of IS elimination (Table 1; Figure 2A). Graft survival in those animals ranged from 29–199 days. One animal demonstrated prolonged graft survival 1,069 days, though it eventually failed to chronic antibody-mediated rejection.

Animals in the experimental group (*n* = 11) received between 1–19 × 10^8^ total nucleated cells/kg comprised of 0.7–16 × 10^6^ CD34^+^ cells/kg and 12–617 × 10^6^ T cells/kg and (Table 1). Renal allograft functional survival rates are shown in Figure 2. Graft survival ranged from 16 days to >4 years in the experimental Kidney Tx + HC cohort. Three of 11 Kidney Tx + HC animals had graft survival greater than 4-years (Figure 2A). The two recipients that achieved mixed chimerism each acheived kidney transplant tolerance greater than 4-years and without GVHD (Figure 2B).

**FIGURE 3:**
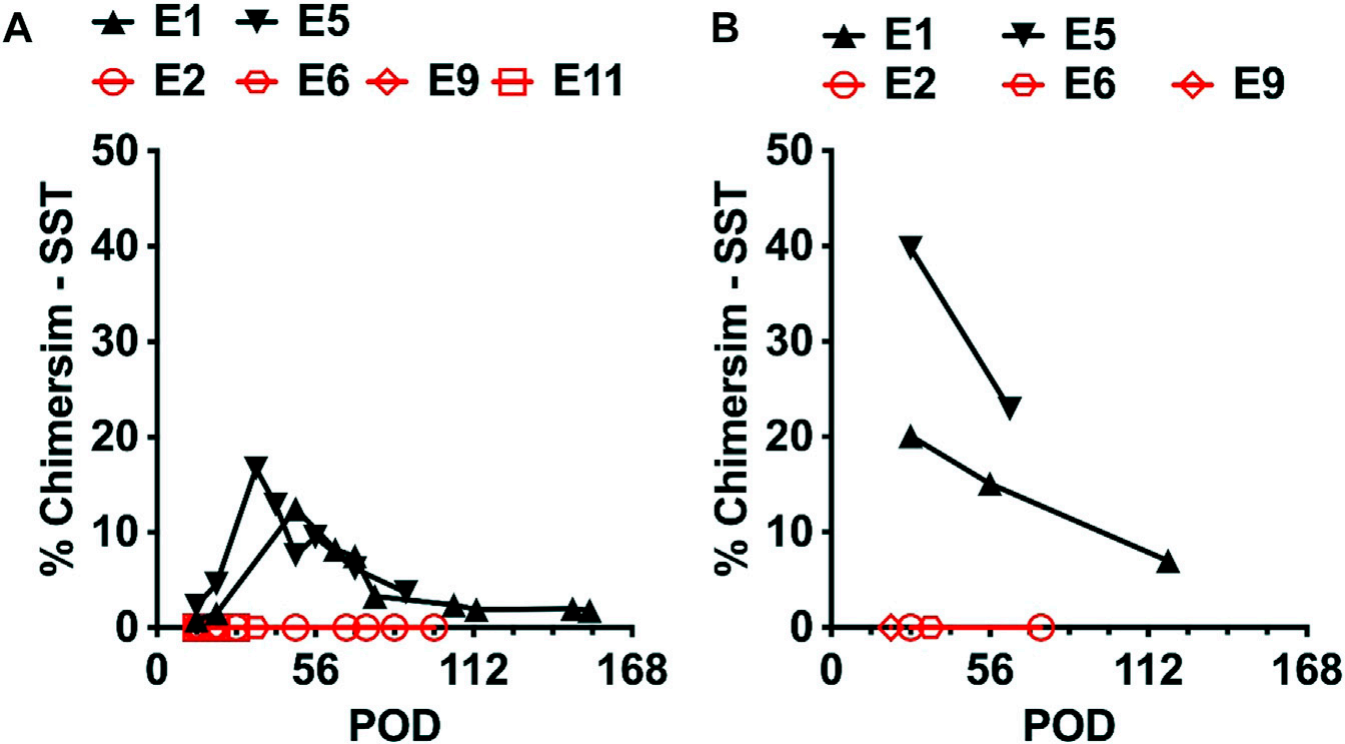
Kinetics of mixed chimerism in the Kidney Tx + HC experimental group (N = 6) as measured by short tandem repeats (STR) in the: **(A)** peripheral blood, and **(B)** bone marrow. Animals E1 and E5 achieved transient mixed chimerism in the peripheral blood and bone marrow. Animals E2, E6, E9 and E11 did not achieve chimerism. Bone marrow was not examined for chimerism in animal E11.

Donor cell chimerism in the experimental group could be ascertained in whole blood using STR analysis in 6 Kidney Tx + HC animals that exhibited greater than 30 days of survival (Figure 3A). Two of the 6 Kidney Tx + HC animals demonstrated transient mixed chimerism. The highest level of engraftment of donor-derived lymphocytes peaked at 20% by day 35, and was lost by 4 months post-induction. Bone marrow analysis revealed that animals with peripheral chimerism also had detectable chimerism within the lymphohematopoietic compartment (Figure 3B). None of the animals in the experimental group developed GVHD.

### TomoTherapy TLI and ATG Conditioning on Leukocyte Depletion

**FIGURE 4:**
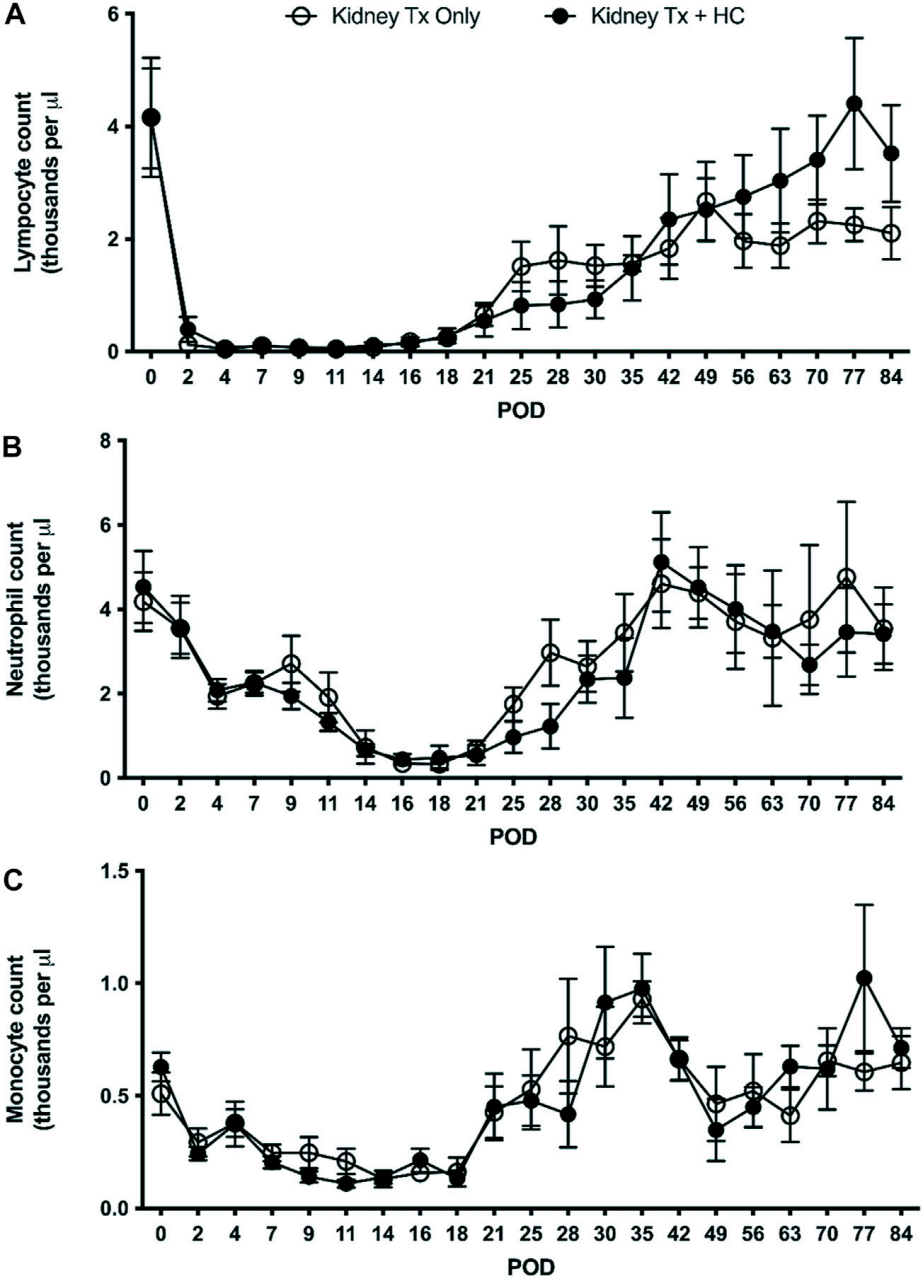
TomoTherapy TLI/ATG effect on blood leukocyte depletion in recipient cell subsets. The number of **(A)** lymphocytes, **(B)** neutrophils and **(C)** monocytes per microliter were determined by complete blood cell count every 2–7 days for Kidney Tx only and Kidney Tx + HC in recipients exhibiting at least 30 days of follow-up.

The efficacy of the tolerance induction protocol on recipient lymphopenia was assessed. TLI/ATG conditioning resulted in prolonged leukocyte depletion (Figures 4A–C) in the peripheral blood. Lymphocyte numbers dropped precipitously by day 2 following initiation of TLI/ATG, reaching the average nadir of <200/µL between days 4 and 7. Recovery, defined as a statistically significant increase in lymphocyte numbers compared to days 4 and 7, was delayed until day 25. Nadir for neutrophils (days 16–18) and monocytes (days 11–14) occurred later compared to lymphocytes, with recovery beginning at approximately the same time as lymphocytes. Upon observation of lymphocyte recovery by day 25, CD8 T cells were first to emerge, followed by NK and B cells, respectively (data not shown). CD4 T cell recovery occured at a similar pace, but only to 30% of pre-transplant values (data not shown).

### TomoTherapy TLI and ATG Conditioning on Immune Modulation

**FIGURE 5:**
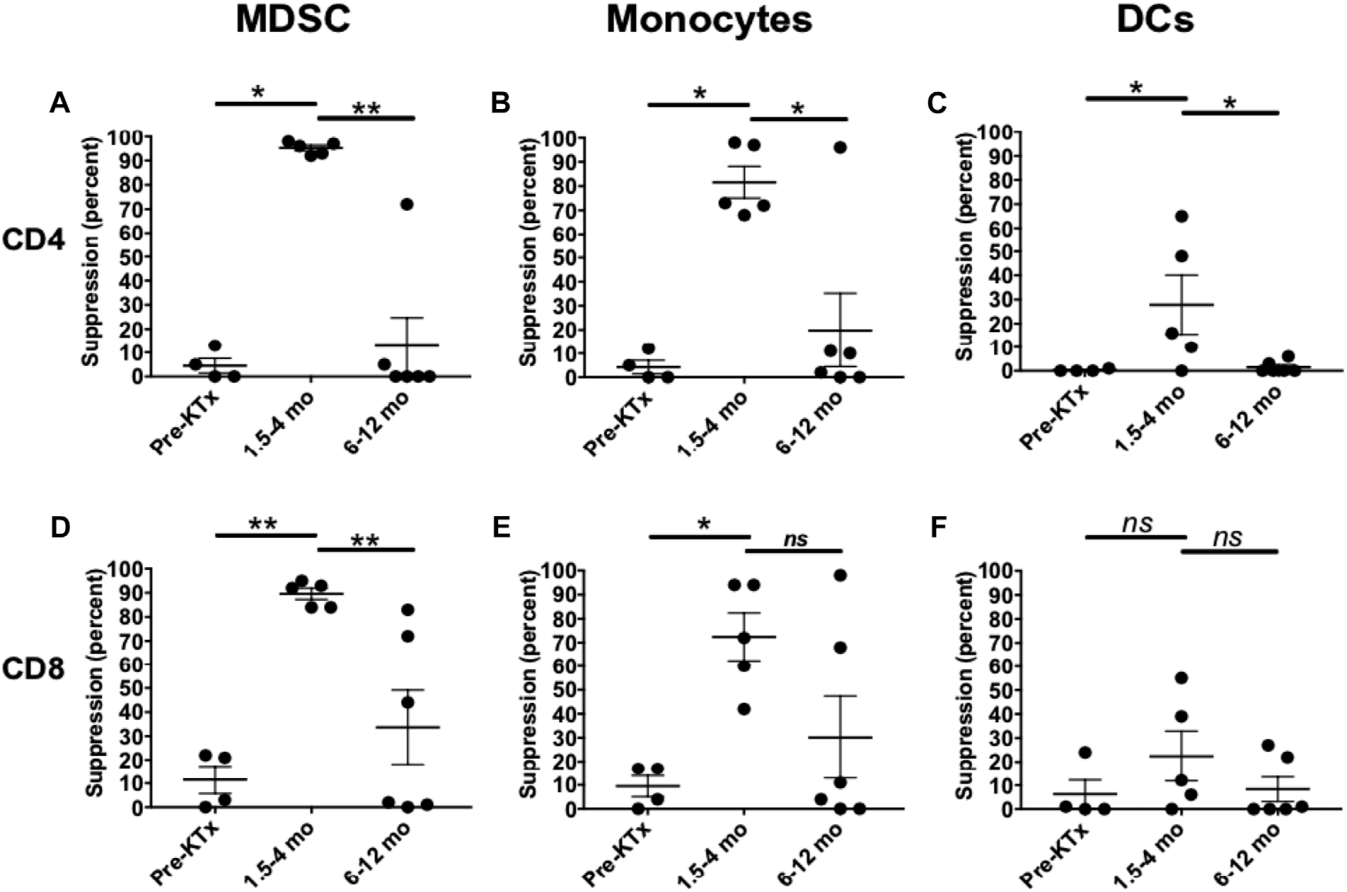
Recipient peripheral blood MDSC and monocytes exhibited immunosuppressive activity following TomoTherapy TLI/ATG tolerance induction. MDSC **(A, D)**, monocytes **(B, E)** and DCs **(C, F)** were isolated at various timepoints and co-cultured with CFSE labelled autologous T cells in the presence of T cell stimulation beads for 5 days. The % suppression of the proliferative response of CD4 T cells **(A–C)** or CD8 T cells **(D–F)** cultured without myeloid cell subsets was determined. Statistical comparisons were made using Student’s t-test, (* = *p* < 0.05; ** = *p* < 0.01).

We examined a unique mechanism of host immune modulation in the rhesus model related to TLI/ATG conditioning that is known to promote chimerism and prevent GVHD in humans and small animals [28, 29]. TLI/ATG-induced suppressive activity of host peripheral blood myeloid derived suppressor cells (MDSCs; Lin^−^CD11b^hi^HLA-DR^lo/-^), monocytes (Lin^−^CD11b^hi^HLA-DR^hi^CD14^hi^) and dendritic cells (DCs; Lin^−^CD11b^lo^HLA-DR^hi^CD11c^+^CD14^−^) on host CD4^+^ and CD8^+^ T cells were tested. In these studies the percent change of CD8 and CD4 T cell subset stimulated proliferation induced by MDSCs, monocytes, DCs, before and at serial timepoints (1.5–3 months, 6–12 months, 12–24 months) after transplantation were measured *in vitro* in several Kidney Tx + HC recipients that received the TomoTherapyTLI/ATG tolerance induction protocol. During the first 4 months post-therapy, both MDSCs and monocytes were able to strongly suppress proliferation of bead-activated autologous CD4 and CD8 T cells (Figure 5). DCs, in contrast, demonstrated less suppressive activity, which was only observed among CD4 T cells populations. Mean percentages of each cell type were compared using the two-tailed Student’s t-test to determine statistical significance. The suppression effects abated in samples drawn from transplant recipients 6–12 months post-transplant as expected and that has been observed in human studies [29].

### Effect of DSA and CMV on Chimerism

**FIGURE 6:**
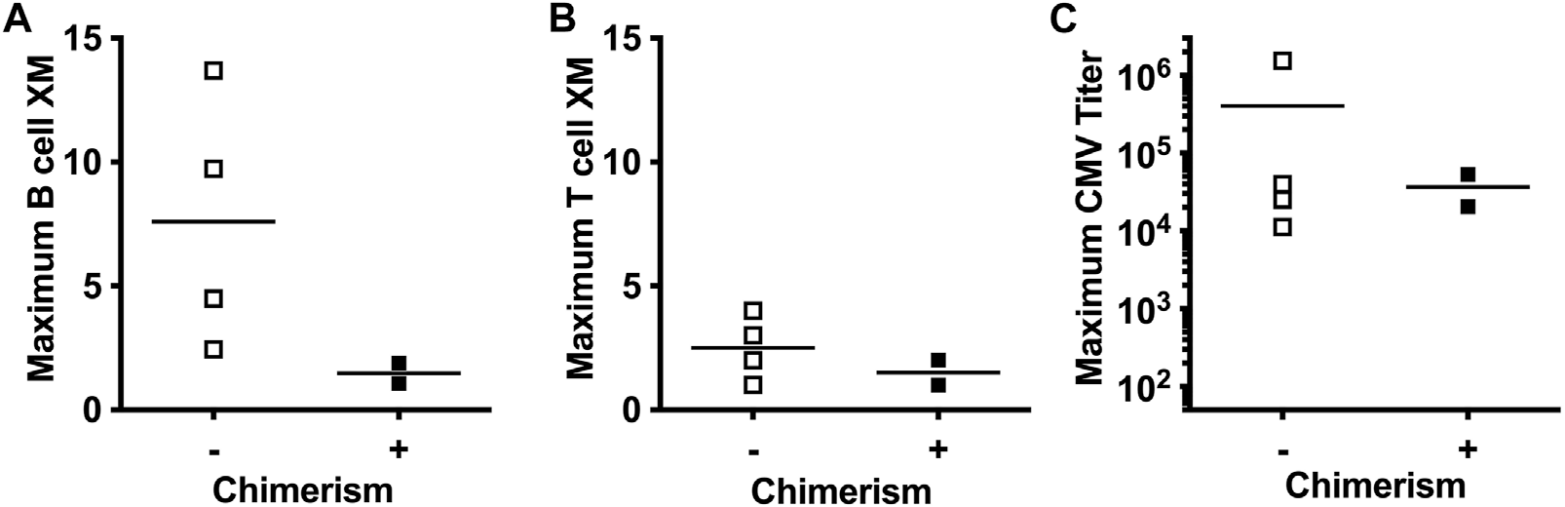
Achieving peripheral donor cell chimerism is associated with absence of generation of antidonor antibody and not CMV reactivation titers. Peak post-transplant anti-donor antibody levels in Kidney Tx + HC recipients with (+) and without (−) Chimerism, as measured by B-cell **(A)** and T-cell **(B)** flow crossmatch. **(C)** Peak post-transplant CMV titers in Kidney Tx + HC recipients with (+) and without (−) Chimerism.

The effect of post-transplant DSA and reactivation of CMV on rates of chimerism were assessed in the six animals in the experimental group that had survival of at least 50 days. Achieving mixed chimerism was associated with an absence of anti-donor specific antibody as measured by B-cell and T-cell flow crossmatch (Figures 6A, B). Conversely, animals that failed to achieve mixed chimerism developed HLA Class II DSA. With respect to CMV reactivation, all animals had detectable increases in peripheral blood CMV titer. No level of CMV titer correlated with either achieving or failing to achieve chimerism (Figure 6C).

### Post-HC Infusion Engraftment Syndrome

**FIGURE 7:**
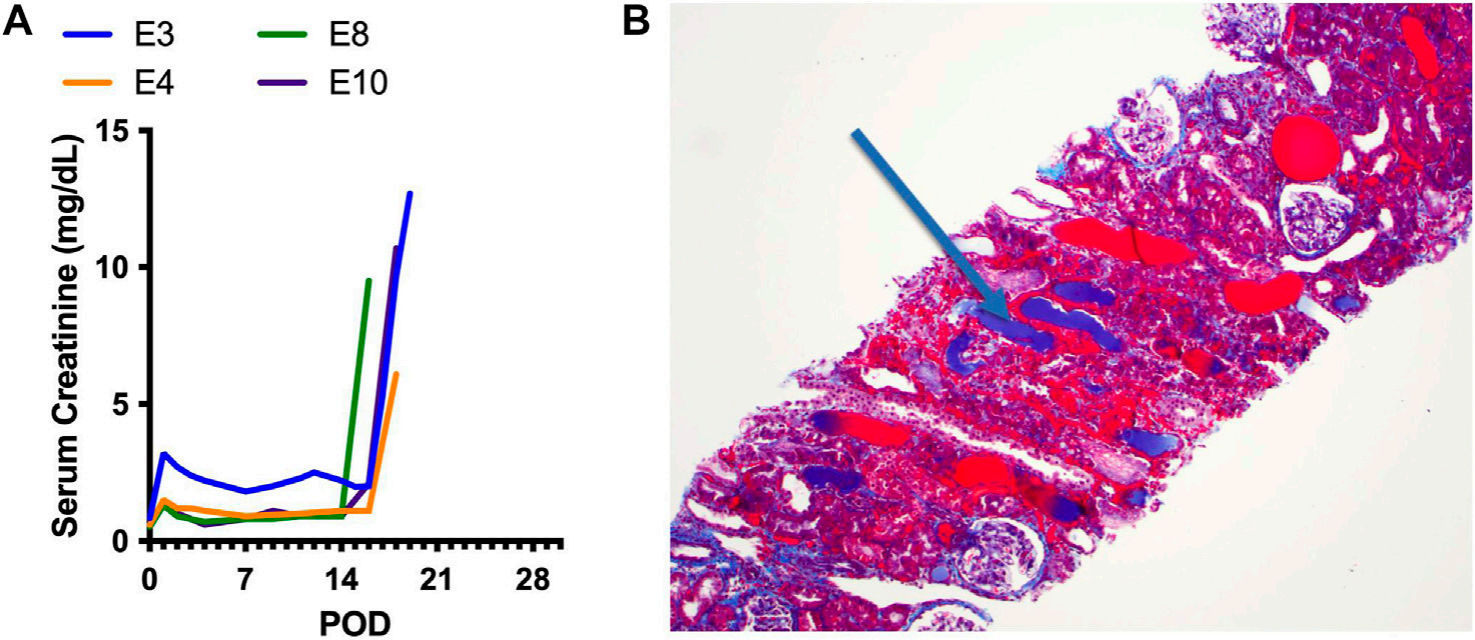
Clinical course of engraftment syndrome. **(A)** Four animals that received a Kidney Tx + HC infusion experienced a rapid increase in creatinine 5–7 days after HC infusion (post-transplant days 16–18). **(B)** Histological analysis of a representative early kidney biopsy demonstrating hyaline casts, interstitial hemorrhage, and tubular necrosis.

Four animals in the Kidney Tx + HC experimental group developed immediate, aggressive and irreversible engraftment syndrome. All 4 animals exhibited rapid acute renal allograft functional decline secondary to non-immune mediated injury (Figure 7A). Histological analysis of the kidneys revealed hyaline casts, interstitial hemorrhage and tubular necrosis (Figure 7B, representative sample).

## Discussion

To achieve kidney transplant tolerance in this rhesus kidney transplant model we used a TLI tolerance induction protocol to create mixed chimerism based on the Stanford clinical protocol [3, 4, 7, 8, 17–19, 30]. TLI tolerance induction protocols in human studies have demonstrated that stable mixed chimerism permits complete elimination of all IS and operational tolerance in well-matched HLA-identical donor/recipient pairs [2–9]. This rhesus model was developed to test the feasibility of TLI condtioning to create mixed chimerism in more disparate 1-haplotype mismatched recipients of combined kidney and HC transplants. To optimize the translational impact of this study we applied a novel TomoTherapy TLI delivery system first developed and reported in primates by this group that also utilized conditioning and maintenance agents approved for human use by the FDA [20]. We then tested the hypothesis that animals achieving chimerism would become operationally tolerant to the kidney allograft for up to 4-years.

The biological underpinings of TLI conditioning to generate chimerism relates to its immunomodulatory effects that have been studied extensively in small animal models and in human pilot studies [13, 29, 31]. In murine studies TLI conditioning was found to activate host MDSCs as characterized by the increased expression of arginase-1, IL-4Rα and programmed death ligand 1 [28]. It was determined that host MDSCs were required for chimerism and tolerance induction in the combined organ and BM transplant model after TLI/ATG conditioning [28]. Depleting this population by monoclonal antibody therapy abrogated chimerism and tolerance while adding back these cells led to restoration of both phenomena. In addition, *in vitro* immunomodulatory properties of the MDSCs demonstrated development of immune suppressive capacity that inhibited the proliferation of host CD8 and CD4 T cell subtypes in response to donor-specific stimulation in an allo-MLR [28]. Importantly, these findings were recapitulated in human subjects involved in TLI/ATG-based chimerism induction studies [29].

In this rhesus model, the three types of myeloid cells (MDSCs, monocytes, and DCs) were detectable in the peripheral blood of the transplant recipients. Importantly, the host MDSCs, monocytes and DCs *in vitro* demonstrated development of immunomodulatory function through suppression of the T cell proliferative response to the presence of potent anti-CD3/CD28 T cell activation beads in culture similar to that previously reported in the human studies [29]. These findings demonstrated for the first time that the TomoTherapy TLI/ATG conditioning methodology applied to the rhesus model was consistent with the immunomodulatory effects achieved with conventional TLI/ATG methodology applied in the human pilot trials [29].

The efficacy of the TomoTherapy TLI/ATG conditioning protocol to generate chimerism had been previously established in rhesus macaques receiving HCs but without the renal transplant [20]. Building on those earlier studies, this study demonstrated that TLI conditioning followed by HC infusion would also generate mixed chimerism in the experimental kidney transplant cohort and proved the hypothesis that chimeric animals would exhibit operational tolerance for over 4-years after IS was withdrawn.

Though our study demonstrated the feasibility of the TLI conditioning protocol to acheive chimerism among the more widely disparate donor/recipients pairs, it proved to be more challenging than what has been observed in well-matched HLA-ID pairs in the human studies, and akin to the challenges in ongoing human 1-haplo matched studies (Stephan Busque, personal communications). However, the observation that operational tolerance was achieved with transient mixed chimerism in this model is consistent with others’ observations that sustained chimerism is not a requirment to successful withdraw maintenance IS without inducing rejection [12, 32].

The chimerism rates in 1-haplo matched rhesus donor/recient pairs was observed much less frequently than that reported in well-matched HLA-identical human recipients. Six experimental animals survived >30 days post-transplant and were evaluable for chimerism, 2 of which achieved transient mixed chimerism in the peripheral blood for up to 112 days. The chimeric monocyte and T-cell subpopulations demonstrated levels similar to those observed in human studies (10%–20%), though at absolute levels less than subjects that achieved stable mixed chimerism. Both also demonstrated transient mixed chimerism in the bone marrow. Interestingly, transient mixed chimerism in the rhesus model was associated with IS-free graft survival indicating that persistent chimerism was not necessarily required for the induction of operational tolerance in this model.

Two potential impediments to achieving mixed chimerism were investigated. These included the development of *de novo* DSA and the occurrence of CMV reactivation. Importantly, the avoidance of DSA correlated with the induction of chimerism. Peak antibody generation against MHC Class II was higher than Class I, indicating that Class II-targeted DSA may be an important barrier to HC engraftment. In this rhesus model all animals experienced CMV reactivation despite prophylactic treatment with anti-virals. However, CMV reactivation was generally not clinically significant and resolved on standard anti-viral therapy without complication. The high frequency of CMV reactivation is an important characteristic of this and other tolerance induction strategies in the rhesus model [33]. Interestingly, the intensity and duration of reactivation, as determined by peripheral blood viral load measurements, were similar in chimeric and non-chimeric animals. This contrasted to results in the Cynologous model indicating that CMV viremia could have a detrimental effect on durable engraftment [34]. This will require further study across the various non-human primate study groups, as this effect could be animal specific and protocol dependent.

The study also demonstrated that four animals in the experimental group experienced rapid and irreversible renal failure with histological findings of interstitial hemorrhage and tubular necrosis consistent with engraftment syndrome. Differences in the donor HC product, as indicated by total number of nucleated cells, T cells, or CD34^+^ cells infused, did not correlate with the development of engraftment syndrome. Furthermore, comparison of the level and duration of leukocyte depletion among all animals within this cohort indicated that the induction agents were consistently effective regardless of the development of engraftment syndrome and did not likely account for the resulting rapid graft loss in such cases. Based on these observations, changes to the maintenance IS regimen, such as intensifying the corticosteroids, represents an opportunity to improve outcomes in future iterations to mitigate occurrences.

Future iterations of the TomoTLI/ATG, non-myeloablative, post-transplant tolerance induction protocol in the rhesus model will require several modifications to enhance the frequency and durability of achieving mixed chimerism. Specifically, modifications to the early maintenance IS regimen will be needed to reduce the risk of engraftment syndrome. In, addition, including a short course of co-stimulatory blockade, such as belatacept, may prevent *de novo* DSA development, as has been observed after adding it to the TomoTherapy TLI protocol in HC-transplant recipients [20].

Another interesting observation was the prolonged survival after IS elimination in one control animal and in an animal in the experimental group that did not achieve chimerism. Eventual kidney failure occurred in both and revealed histologic findings of acute and chronic antibody-mediated rejection in addition to moderate interstitial fibrosis and tubular atrophy. All the recipients received TLI/ATG. It is known from human studies that TLI/ATG added to a conventional immunosuppression protocol in high-risk renal re-transplant recipients has been associated with prolonged survival and development of donor-specific hyporesponsiveness [35–37]. Though the numbers are small, by trend, it appears that transient mixed chimerism is sufficient for prolonged tolerance, but not an absolute necessity. An occasional recipient receiving ATG/TLI induction does achieve prolonged survival after immunosuppression has been weaned off in this model.

In summary, there were several important insights gained from this model. Promising results were demonstrated in this tolerance induction protocol applied to 1-haplotype matched donor/recipient pairs that permited the elimination of all IS without rejection or GVHD while maintaining 4-years of operational tolerance of the renal allograft. Opportunities to enhance the results were also presented. Eliminating engraftment syndrome and improving the rate of mixed chimerism will be crucial to the extension of this protocol to more disparate MHC barriers. Additionally, mechanistic studies will need to be continued and expanded in order to elucidate the immunologic mechanisms underlying mixed-chimerism based tolerance, so as to leverage potential targets and manipulations for future induction strategies. Knowledge gained through this rhesus tolerance induction model could possibly have direct relevance to a wide variety of donor transplants including deceased donation cases, which would dramatically expand the implementation of tolerance induction protocols beyond the limited pool of living related pairs.

## Data Availability

The original contributions presented in the study are included in the article/Supplementary Material, further inquiries can be directed to the corresponding author.

## Abbreviations

ATG, Anti-thymocyte globulin; DC, Dendritic cells; DSA, Donor specific antibody; GVHD, Graft-versus-host disease; TomoTherapy TLI, Helical TomoTherapy-based total lymphoid irradiation; HC, Hematopoietic cell; IS, Immunosuppression; MLR, Mixed lymphocyte reaction; MMF, Mycophenolate Mofetil; MDSC, Myeloid derived suppressor cells; NIAID, National Institute of Allergy and Infectious Disease; NIH, National Institute of Health; POD, Post-operative day; RhCMV, Rhesus Cytomegalovirus; STR, Short tandem repeats; TLI, Total lymphoid irradiation; WNPRC, Wisconsin National Primate Research Center.
