# Supplementary material for: Helical TomoTherapy Total Lymphoid Irradiation and Hematopoietic Cell Transplantation for Kidney Transplant Tolerance in Rhesus Macaques
Source: Transpl Int. 2023 Jun 22;36:11279. doi: 10.3389/ti.2023.11279 (PMC10324513; doi:10.3389/ti.2023.11279)
Supplement: Supplementary file 1 [file DataSheet1.pdf]

Helical TomoTherapy Total Lymphoid Irradiation and Hematopoietic Cell Transplantation for Kidney Transplant Tolerance in Rhesus Macaques

Dixon B. Kaufman[1], Lisa Forrest[2], John Fechner[1], Jennifer Post[1†], Jennifer Coonen[3], Lynn D. Haynes[1], W. John Haynes[1], Neil Christensen[2], Weixiong Zhong[4], Christopher J. Little[1], Anthony D'Alessandro[1], Luis Fernandez[1], Kevin Brunner[3], Kent Jensen[5], William J. Burlingham[1], Peiman Hematti[4], Samuel Strober[5†]

[1]University of Wisconsin, Department of Surgery, Madison, WI 53792

[2]University of Wisconsin, School of Veternary Medicine, Madison, WI 53792

[3] Wisconsin National Primate Research Center, University of Wisconsin, Madison, WI 53711

[4]University of Wisconsin, Department of Medicine, Madison, WI 53792

[5]Stanford University, Department of Medicine, Palo Alto, CA

[†]Deceased

Corresponding author:
Dixon B. Kaufman, MD, PhD, FACS
Department of Surgery, Division of Transplantation
School of Medicine and Public Health, University of Wisconsin-Madison
H4/722, Clinical Sciences Center, 600 Highland Ave.
Madison, WI 53792
T: 608-265-6471; F: 608-262-6280
kaufman@surgery.wisc.edu

Running title: Kidney Transplant Tolerance in Rhesus Macaques
Keywords: Kidney transplantation, tolerance induction, chimerism, hematopoietic cells, TomoTherapy

Research reported in this publication was supported by the National Institute of Allergy and Infectious Diseases of the National Institutes of Health as part of the Nonhuman Primate Transplantation Tolerance Cooperative Study Group under Award Number U01AI102456 (PI, Kaufman) and T32AI25231 (PI, Kaufman). The content is solely the responsibility of the authors and does not necessarily represent the official views of the National Institutes of Health. Rhesus anti-thymocyte globulin reagents used for these studies was provided by the NIH Nonhuman Primate Reagent Resource (P40 OD028116, U24 AI126683).

Supplemental Materials

Animals, MHC determination of donor-recipient pairs, and Peri-operative Antibiosis.

All animals were negative for Mycobacterium tuberculosis, Macacine herpesvirus 1 (herpes B virus), simian T-lymphotropic virus, simian retrovirus type D, and simian immunodeficiency virus.

MHC Class I and Class II typing of recipient and donor animals were performed by the WNPRC Genetics Services Unit as described previously[21,22]. Briefly, genomic DNAs isolated from whole blood samples served as templates for PCR with primers that bind conserved sequences flanking the highly polymorphic peptide binding domains encoded by exon 2 of Mamu-A, -B and -DRB loci. After purification with AMPure XP beads and pooling, these amplicons were sequenced on an Illumina MiSeq instrument and the resulting sequence reads were mapped against a custom database of Mamu-A, -B and -DRB sequences. Ancestral Mamu-A, -B and DRB haplotypes were inferred based on these deep sequencing results for each animal. These MHC haplotyping results, along with pedigree analysis, were used to determine appropriate donor/recipient pairs for each transplant group.

Most animals received Valgancyclovir (5 mg/kg BID) through day 90 or until evidence of rhCMV reactivation in the plasma was below the level of assay detection. Animals also received a peri-operative course of antibiotics during immunosuppression, typically Cefazolin IM BID for 2 weeks, Cephalexin PO BID for 2 weeks, then Sulfamethoxazole + trimethoprim (TMS) PO BID for 90 days. Ceftriaxone was indicated in the event of neutropenia.

Helical Tomotherapy for Total Lymphoid Irradiation

Computed tomographic simulation of the entire body in contiguous 2.5 mm slices was performed prior to TLI with a GE Lightspeed scanner (GE Healthcare, Waukesha, WI) at 200 mAS and 120 kVp. TLI was planned and delivered by imaged-guided, intensity modulated

helical tomotherapy (Tomotherapy Hi-Art II, Accuray Inc, Sunnyvale CA). A PTV margin of 4mm was isotopically expanded from the lymphoid GTV. The lymphoid GTV was prescribed 12 Gy in 10 fractions to 75% of the volume. Organs at risk included the left and right lungs, liver, bladder, colon, rectum, small bowel, bladder, skin and brain. A summated structure of all non-target tissues was created to determine total normal tissue dose. Dose control structures were created and utilized as required to control dose within the normal tissues. Iterative inverse planning was used to minimize the dose to these organs at risk. Offline adaptive planning was used to account for change in body weight or composition during the radiation delivery.

Chimerism measurement details

Polymorphisms of short tandem repeats (STR) throughout the genome were used to distinguish products derived from donor or recipient origin. The area under all electropherogram peaks were measured from each reaction (DAx data acquisition software; Van Mierlo Software Consultancy, Eindhoven NL). A comparison of the peak areas for each allele from three replicate reactions (at a minimum of three loci) were made with five standard dilution mixtures of donor and recipient DNA purified prior to transplant.

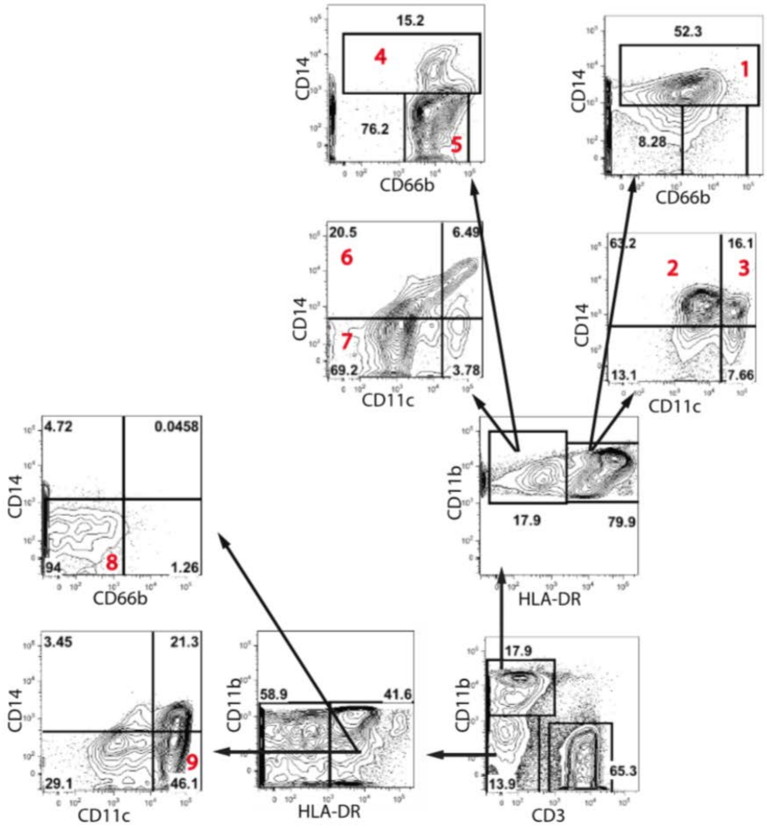

Figure S1. Two color flow cytometric analysis of staining of myeloid cell subsets from the peripheral blood in rhesus macaques. Panels show receptor expression after gating out T cells, B cells and NK cells. Boxes show receptor expression on different myeloid cell types. Anti-CD20, -NKp80, and -CD3 mAbs were used to gate out Lin+ cells.The 3 types of myeloid cells were defined with anti-CD11b, CD66b, DR, CD14 and CD11c mAbs. These included Lin-CD11b^{hi}DR+CD14+ monocytes that had CD11c^{int} and CD11c^{hi} subsets as shown in boxes 2 and 3. The monocytes showed intermediate staining for CD66b (a granulocytic marker, box 1). Most MDSCs expressed the Lin-CD11b^{hi}DR-CD14-CD11c^{lo} pattern shown in box 7. Some cells were CD66b^{hi} (box 5), and were predominantly granulocytic MDSCs. A minority of CD14+ monocytic MDSCs are shown in boxes 4 and 6. Myeloid DCs expressed the CD11b^{lo}DR+CD11c^{hi} CD14- phenotype (box 9). Almost all of these cells were CD66b-.
